# Differential Impact of Social Isolation and Space Radiation on Behavior and Motor Learning in Rats

**DOI:** 10.3390/life13030826

**Published:** 2023-03-18

**Authors:** Austin M. Adkins, Emily M. Colby, Alea F. Boden, Justin D. Gotthold, Ryan D. Harris, Richard A. Britten, Laurie L. Wellman, Larry D. Sanford

**Affiliations:** 1 Sleep Research Laboratory, Norfolk, VA 23507, USA; 2 Center for Integrative Neuroscience and Inflammatory Diseases, Norfolk, VA 23507, USA; 3 Department of Pathology and Anatomy, Norfolk, VA 23507, USA; 4 Radiation Oncology, Eastern Virginia Medical School, Norfolk, VA 23507, USA

**Keywords:** sensorimotor performance, sleep, space radiation, social isolation, stress resilience, stress vulnerability

## Abstract

Future missions to Mars will expose astronauts to several physical and psychological challenges, including exposure to space radiation (SR) and periods of social isolation (SI). Each of these stressors, in addition to mission demands, can affect physical and mental health and potentially negatively impact sleep. The effects of inflight stressors may vary with duration and time course, may be additive or compounding, and may vary with individual differences in stress resilience and vulnerability. Determining how individual differences in resilient and vulnerable phenotypes respond to these mission-related stressors and their interactions with sleep will be crucial for understanding and mitigating factors that can impair performance and damage health. Here, we examined the single and compound effects of ground-based analogs of SI and SR on sensorimotor performance on the balance beam (BB) in rats. We also assessed emotional responses during testing on the BB and assessed whether sensorimotor performance and emotion varied with individual differences in stress resiliency using our established animal model in which stress produces different effects on sleep. Results showed differential motor performance and emotion in the BB task between SI and SR, and these varied based on resilient and vulnerable phenotypes. These findings demonstrate that identifying individual responses to stressors that can impact sensorimotor ability and behavior necessary to perform mission-related tasks will be of particular importance for astronauts and future missions. Should similar effects occur in humans, there may be considerable inter-individual variability in the impact that flight stressors have on the mental health of astronauts and their ability to perform mission-related tasks.

## 1. Introduction

During the long durations of the proposed NASA Mars missions, astronauts will be exposed to several physical and psychological challenges, including exposure to space radiation (predicted~13 cGy/Yr (SR)) and periods of social isolation (SI). Individually, SI and SR have been reported to alter behavior [1,2], spatial working memory [2], and sensorimotor functions [3,4,5]. However, the synergistic effects of SI and SR combined are all but unknown. Astronauts must maintain the ability to conduct tasks that require both gross movements and fine motor coordination, which could include performing basic routine assignments as well as potentially performing mission critical repairs. Any of these tasks requires proper processing of sensory information, cognitive integration and decision making, and performing the appropriate motor task (i.e., sensorimotor integration).

Stress can disrupt sleep [6,7,8] and can have negative effects on tasks that require attention, decision making, and memory, which impedes performance [9] and the latter of which can also be impacted by disrupted sleep [10,11,12]. An additional important consideration for understanding the effects of inflight stress is that both humans and animals can be differentially resilient or vulnerable to the effects of stress [13,14,15]. Studies in humans have characterized the protective effect of stress resilience on high- and low-strain conditions [16], and previous studies by our lab have shown that circulating BDNF [17] and stress-induced changes in sleep [18,19] can vary between resilient and vulnerable rats. Therefore, the effects of SI and SR on performance metrics may also change based on differences in stress resilience and vulnerability.

In our work, we focused on sleep to delineate differential stress responses. Vulnerable rats show decreases in rapid eye movement sleep (REM) after training with inescapable footshock, whereas resilient rats show no decreases, or even increases, in REM [18,19]. The vulnerable and resilient phenotypes are also differentially responsive to other stressors (e.g., novel chamber [17] and simulated space radiation [20]) indicating the differences are not limited to footshock stress. At a conceptual level, animals that sleep normally or have increased sleep after stress have a more adaptive response that is less likely to lead to long-term stress-related problems than do animals with disrupted sleep. This rationale is consistent with literature that emphasizes roles for disrupted sleep in stress-related pathology [21,22,23] and with a restoration of homeostasis as the stress responses end [24].

Determining how individual astronauts respond to mission-related stressors, and how inflight stress can impair performance and health, is essential for our understanding and necessary to ensure successful and safe missions. In this study, we used ground-based analogs of SI and SR to examine the single and compound effects of SI and SR on motor learning sensorimotor performance on the balance beam (BB) task in rats. We also assessed emotional responses during testing on the BB and assessed whether learning ability, sensorimotor performance, and emotion varied with resilient and vulnerable phenotypes using our established animal model of stress resilience and vulnerability [18,19].

## 2. Materials and Methods

### 2.1. Subjects

Male, outbred, Wistar strain rats (8–9 months old at time of study) were obtained from Hilltop Lab Animals, Inc. (Scottdale, PA, USA) and randomly assigned to SI (visual barriers between cages, *n* = 21) or individual housing (as a Control group, *n* = 20). SI began at least eight weeks prior to experimentation and was maintained throughout the study. Separate groups of rats received a single dose of SR (15 cGy simplified 5-ion GCRsim, Brookhaven National Laboratory; Long Island, NY, USA) treatment and were randomly assigned to either individual housing (*n* = 15) or to SI (*n* = 16).

A Sham group traveled with SR-treated groups as a control to account for any potential negative effects on the animals’ sleep and behavior caused by the transit required for the experiment and either individually housed (*n* = 5) or subjected to SI (*n* = 3). No significant differences in behavior or sleep parameters between animals maintained in house and Sham groups (either Control or SI) were observed (Supplementary Figures S1 and S2). Therefore, Sham rats were incorporated into respective Control or SI groups (depending on treatment received) for subsequent analyses moving forward (updated sample sizes: *n* = 25 and *n* = 24 for Control and SI groups, respectively). Food and water were available *ad libitum*. Housing rooms were kept on a 12:12 light:dark cycle, and ambient temperature was maintained at 24.5 °C ± 0.5°. All procedures were conducted in accordance with the National Institutes of Health Guide for the Care and Use of Experimental Animals and were approved by Eastern Virginia Medical School’s Institutional Animal Care and Use Committee (Protocol#: 19-018).

### 2.2. Surgery

At least three weeks prior to behavioral testing, all rats were implanted intraperitoneally with telemetry transmitters (ETA F10, Data Sciences International; Minneapolis, MN, USA) for recording EEG activity, gross body activity, and whole-body temperature. Leads from the transmitter body were led subcutaneously to the head, and the free ends were placed into holes drilled in the dorsal skull to allow for recording. All surgeries were conducted under isoflurane (inhalant: 5% induction; 2–3% maintenance) anesthesia. Ibuprofen (30 mg/kg, oral) was continuously available in each animal’s drinking water for 24–48 h pre-operatively and for a minimum of 72 h post-operatively for pain relief. All animals received prophylactic procaine penicillin (22,000–100,000 IU/kg), gentamicin (5–8 mg/kg), and dexamethasone (0.5–2 mg/kg) subcutaneously on the day of surgery.

### 2.3. Balance Beam Procedures

BB assesses fine and gross motor function through the animals’ ability to traverse the beam. We utilized a modified version of the BB paradigm based on previous studies [25,26,27] where rats were trained to traverse a 2 cm wide by 1 m long beam suspended 90 cm over soft padding for 5 trials a day for 7 consecutive days (35 trials in total). Motivation to cross was provided by white noise proximal to the start platform and the presence of a treat (Lucky Charms^®^ cereal) in the goal box. The beam was marked lengthwise from 1–3 in equivalent sections from the goal box. Training began on at the start of the fifth hour of lights on. For training, the animal was placed at position 1 (closest to the goal box) on the beam. If they succeeded in entering the goal box, the next trial began at the next farthest location (i.e., position 2, then position 3). If they succeeded at position 4 (the start platform), the animal remained at position 4. The trial was considered over when the rat either reached the goal box, fell off the beam, or 2 min elapsed. If the rat fell or timed out, they were moved to one location closer to the goal box. If this occurred as position 1, the animal remained at position 1. Between each trial, the rats were returned to their home cage for a 5 min rest period. Visual barriers between cages were maintained for rats in the SI and concurrent SI and SR exposure (DFS) groups for all testing periods.

Criterion for successful learning was determined by an individual animal reaching the goal box starting at position 4. The learning period (LP) was considered to be all trials up to the first trial that the animal reached criterion (position 4). Once criterion was reached, subsequent trials were considered to be the post-learning period (PLP). During the PLP, animals’ performance was recorded as meeting criterion (success from position 4), as well as “pass” for success from any other position (1–3). However, regardless of reaching criterion or not, animals were tested for the entirety of the 7 days for assessment of learning and the ability to maintain performance rates following learning. Latency to complete the task or fall off the beam was recorded in seconds (s). The average number of trials to reach criterion per group was recorded. Percent of animals that learned (reached criterion) in each treatment group was calculated based on the number of animals that were successful from position 4 over the total number of animals in the group (%Learned = # of Animals that Reached Criterion/Total # of Animals within the Group × 100). Percent pass rates for each position during LP and PLP were calculated based on the number of trials where animals within each treatment group reached the goal box from a respective position (1, 2, or 3) over the total number of trials that animals spent at that position (%Pass Rates = # of Passed Trials at a Position/Total # of Trials at a Position × 100). Overall percent pass rates during LP and PLP were calculated based on the number of successful trials that animals within each treatment completed over the total number of trials available during the BB task (i.e., 35) (Overall %Pass Rates = # of Successful Trials/Total # of Trials × 100). Criterion success rates were measured based on an animals’ entering of the goal box after successfully crossing from position 4. Percent success was calculated for the animals within a group that reached criterion based on the number of successful trails from position 4 over the total number of trials attempted at position 4 (%Success = # of Successful Trials at Criterion/Total # of Trials at Criterion × 100).

To further delineate potential differences in the groups, we assessed additional behaviors observed during each trial, which we categorized as exploratory, disequilbrium, fear, and maintenance behaviors. Exploratory behaviors included observations of vacillation, spinning, and rearing. Disequilibrium behaviors included compromised ambulation, impaired gait, balance issues, and disorientation. Fear behavior was identified as observations of freezing. Maintenance behavior included grooming.

### 2.4. Determination for Resilient and Vulnerable Subgroups

Following completion of BB testing, the rats were trained in a conditioned fear paradigm and classified as either resilient or vulnerable based on percent change in REM amounts following footshock training shock compared to baseline (%Change = Total REM post-shock/Total REM Baseline × 100) using an established procedure [18,19]. Briefly, following shock, vulnerable rats were determined based on a 50% or greater decrease in REM during the first 4 h of recording compared to baseline. Resilient rats were determined based on smaller decreases (≤50%), no change, or increases in REM compared to baseline. Sleep recording occurred within the same room the animals were housed. For recording, individual cages were placed on a telemetry receiver, and the transmitter was activated with a magnet. When the animals were not on study, the transmitter was inactive. Telemetry signals were processed and collected and then visually scored by a trained individual blinded to treatment condition in 10 s epochs using the Neuroscore sleep analysis program (Data Sciences International; Minneapolis, MN, USA). Epochs were scored based on EEG and whole-body activity. Specifically, REM was scored by the presence of high-frequency, low-amplitude EEG with the presence of theta rhythms and body inactivity.

### 2.5. Statistical Analyses

Percent change in sleep data was analyzed using an ordinary one-way analysis of variance (ANOVA) test. Tukey’s post hoc multiple comparisons test was performed when indicated by a significant ANOVA.

Performance data on the BB task was either analyzed with an ordinary one-way ANOVA (without phenotype separation) or a two-way mixed factor ANOVAs with Phenotype(resilient and vulnerable) as between factors and Test Day as a within subject factor when appropriate. Tukey’s post hoc multiple comparisons test was performed when indicated by a significant ANOVA. All ANOVAs were generated using GraphPad PRISM software (Version 9.4.1).

## 3. Results

**Figure 1:**
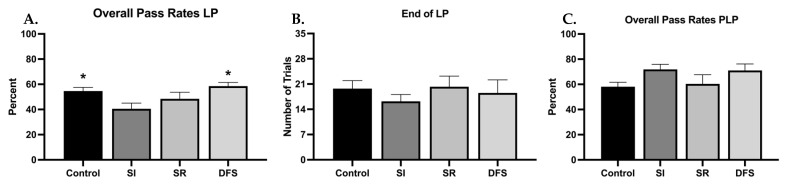
**Lack of Phenotypic Separation Reveals Minimal Differences in the Balance Beam Task During the Learning Period (LP) and Post-Learning Period (PLP).** Graphs plotting differences between groups for (**A**) the percent pass rates ± SEM of each group during the LP, (**B**) the average number of trials ± SEM needed to end the LP for each group, and (**C**) the percent pass rates ± SEM of each group during the PLP. Compared to SI: * *p* < 0.05.

**Figure 2:**
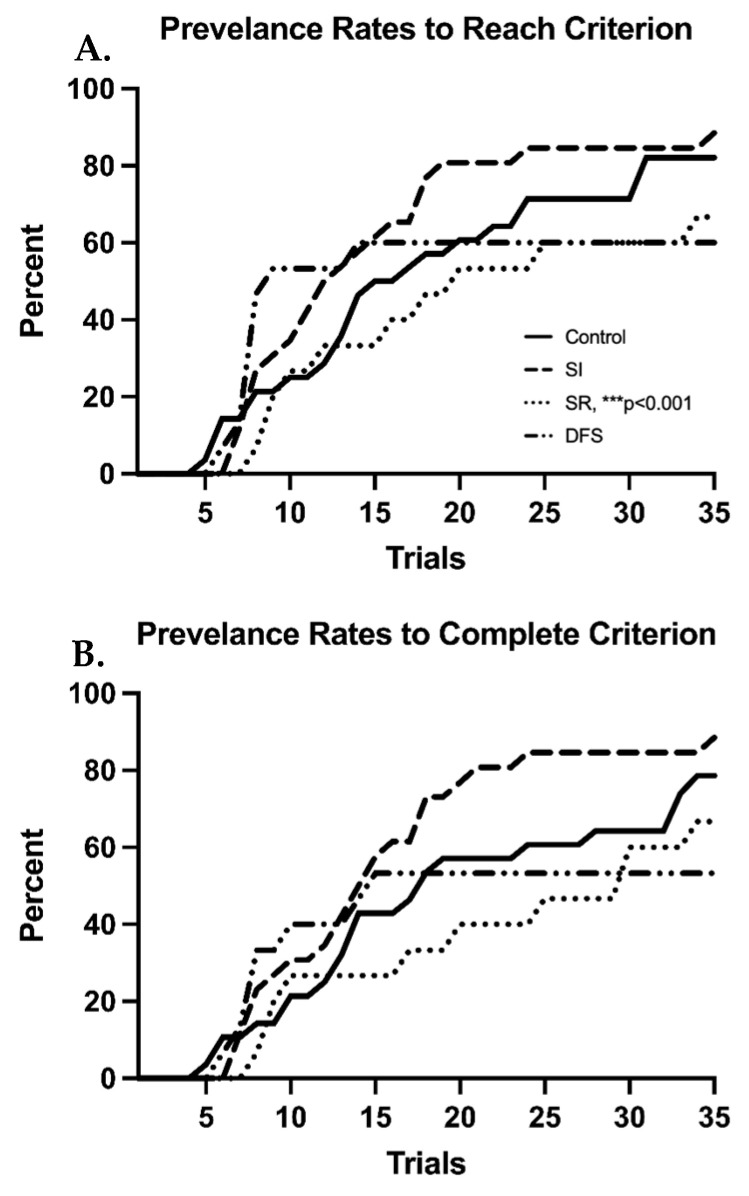
**Irradiation Reduces the Number of Animals Able to Reach Criterion.** Line graphs showing the percentage of animals within each group that (**A**) attempted to cross the beam from position 4 in a given number of trials and (**B**) animals that successfully completed criterion (success from position 4) in a given number of trials. Compared to SI: *** *p* < 0.001 for both graphs.

There were minimal differences between each treatment group’s ability to learn the BB task when resilient and vulnerable phenotypes were not considered separately (Figure 1A–C). However, one-way ANOVA revealed significant differences between groups (F_3,77_ = 4.002; *p* = 0.01), and Tukey’s post hoc revealed the SI group had worse overall pass rates during the LP compared to the other groups (*p* < 0.05 compared to Control and DFS) (Figure 1A). Despite this, all groups ended their LP in in a similar number of trials (F_3,77_ = 0.5610; *p* = 0.6424) (Figure 1B). When measuring performance rates of animals that reached criterion, thereby ending their LP, overall pass rates during the PLP were not significantly different (F_3,78_ = 2.491; *p* = 0.0664) (Figure 1C). Percentage plots showing the time course of each group’s average number of trials to reach and complete criterion demonstrate clear deficits in the SR and DFS groups compared to the Control and SI groups (Figure 2A,B). By the end of the BB task, 66.7% of the total animals in the SR group (*p* < 0.001 compared to SI) and 60% of the animals in the DFS group were able to reach criterion. The same percentage of SR animals also could successfully complete a trial into the goal box from criterion (*p* < 0.001 compared to SI), but only 53.3% of the animals in the DFS group could successfully complete a trial from criterion. Contrastingly, 88.5% of the total animals in the SI group were able to reach criterion and successfully complete a trial from criterion by the end of the BB task. The Control group demonstrated 82.1% of the total animals within the group reach criterion by the end of the BB task, but only 78.6% of the group successfully completed a trial from criterion. However, this was not due to any observable impairment, but once these animals learned, they engaged in more off-task behaviors unrelated to completing the BB task.

To further delineate potential performance differences across groups, we assessed additional categories of behavior (Exploratory, Disequilbrium, Fear, and Maintenance) during the LP and PLP (Table 1 and Table 2, respectively). Overall, during the LP, the Control group exhibited more exploratory and maintenance behaviors unrelated to completing the BB task. The SI, SR, and DFS groups exhibited instances of disequilibirum, which resulted in increased difficulty to complete the BB task. Furthermore, the SR and DFS groups also exhibited fear behaviors during the task, which, in certain intences, led to an apparent unwillingness to attempt the task. During the PLP, the behaviors exhibited within each group persited or increased. Thus, the behaviors in the Control group were categorically different from those of the SI, SR, and DFS groups in ways that standard assessments on the BB do not detect.

**Figure 3:**
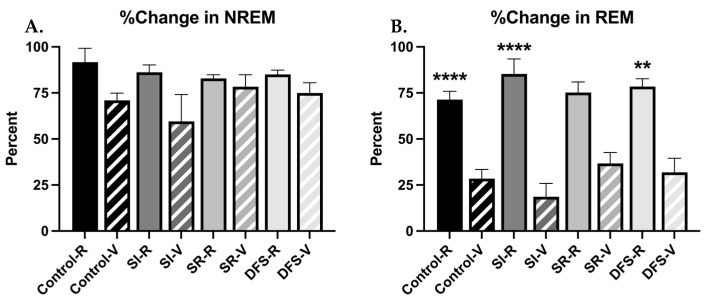
**Vulnerable Animals Showed Decreased REM after Shock Compared to Resilient Animals.** Graphs plotting the first 4 h of sleep recording during baseline and after shock training (post-shock) of resilient (R) and vulnerable (V) animals within each treatment group for (**A**) the percent change ± SEM in NREM and (**B**) the percent change ± SEM in REM from baseline after shock. Significant differences compared to V: ** *p* < 0.01, **** *p* < 0.0001.

Rats were divided into resilient and vulnerable groups based on REM responses to footshock stress as described in a previously published work [18,19]. This provided distinct resilient and vulnerable responses and forms the basis for the remaining comparisons in this report. Overall, no differences in total REM or NREM sleep between resilient and vulnerable animals maintained in house and Sham groups were observed (F_3,46_ = 2.297; *p* = 0.0901 and F_3,46_ = 0.9601; *p* = 0.4196, respectively) (Supplementary Figure S1A,B), so Sham rats were incorporated into respective Control or SI groups (depending on treatment received and phenotype) for subsequent analyses. The following comparisons were made with these new groups moving forward: differences between Control resilient (*n* = 16) and treatment resilient groups (SI, *n* = 16; SR, *n* = 13; and DFS, *n* = 11), differences between Control vulnerable (*n* = 10) and treatment vulnerable groups (SI, *n* = 8; SR, *n* = 2; and DFS, *n* = 3), and differences between resilient and vulnerable phenotypes within each group. Differences in resilient and vulnerable phenotypes were observed in the effects of shock on sleep (Figure 3A,B). When measuring % change in total NREM and REM amounts following shock compared to baseline within the first 4 h of recording, ANOVA revealed significant differences between groups (F_7,68_ = 2.392; *p* = 0.03), but post hoc analysis did not reveal significant differences observed in the % change of NREM, though vulnerable animals within each group did show slight decreases in NREM compared to resilient animals. ANOVA revealed significant differences between Groups when measuring percent change in REM (F_7,68_ = 14.29; *p* < 0.0001), and Tukey’s post hoc revealed Control (*p* < 0.0001), SI (*p* < 0.0001), and DFS (*p* < 0.01) vulnerable rats exhibited a greater percent decrease in total REM sleep following shock compared to resilient rats. This was not significant within the SR group due to a low sample size within the vulnerable phenotype (*p* = 0.232) (Figure 3B).

**Figure 4:**
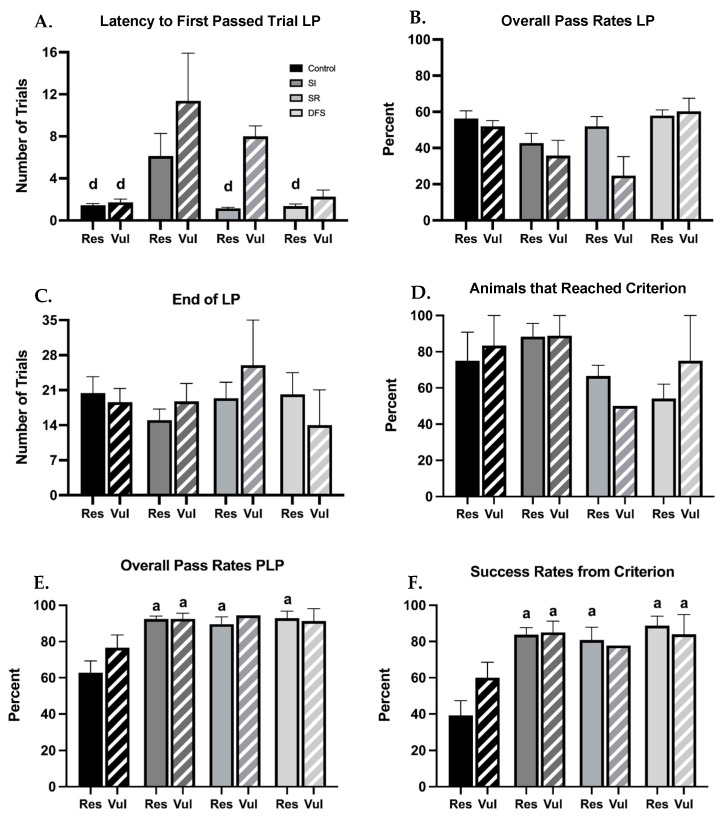
**Differences in the Ability to Complete the Balance Beam Task are Seen with Phenotype Separation During the Learning Period (LP) and Post-Learning Period (PLP).** Graphs plotting differences between resilient (Res) and vulnerable (Vul) phenotypes within each treatment group for (**A**) the average number of trials ± SEM for a group to complete their first successful crossing or “pass” (from position 1), (**B**) the percent pass rates ± SEM of each group during the LP, (**C**) the average number of trials ± SEM needed to end the LP for each group, and (**D**) the percent of animals ± SEM that reached criterion (position 4). (**E**) The percent pass rates (regardless of position) ± SEM of each group during the post-learning period (PLP), and (**F**) the percent success rates of animals ± SEM from criterion (position 4). Compared to Control: a, *p* < 0.05; SI: d, *p* < 0.05.

Following separation of resilient and vulnerable phenotypes within each treatment group, clear differences in performances in the BB sensorimotor tasks were also observed. When measuring the number of trials to complete their first successful crossing or “pass” from position 1, ANOVA revealed significant differences between Groups (F_1,73_ = 4.452; *p* = 0.03) and Treatment (F_3,73_ = 7.299; *p* < 0.001) but not between Group × Treatment interaction Rats subjected to SI required a higher number of trials to complete their first successful pass (from position 1) compared to other groups, and this was amplified in the SI vulnerable group (*p* < 0.01 for all significant comparisons) (Figure 4A), The SR resilient and DFS resilient and vulnerable groups completed their first successful crossing in a similar number of trials as the Control group. The initial delay in the SI group did not appear to be a persisting impediment, as once learned, they appeared to have no further deficits and performed similarly to the Control group, regardless of the resilient or vulnerable phenotype. Despite no differences in percent pass rates between the treatment group or phenotype during the LP being observed (*p* > 0.24) (Figure 4B), and all resilient and vulnerable animals within each group ending their LP within a similar number of trials (*p* > 0.91) (Figure 4C), differences in the percentage of animals within each group that were able to appropriately perform the BB task and reach criterion were found. SR and DFS groups had the lowest percentage of animals to reach criterion, and this was different between resilient and vulnerable phenotypes (SR: ~75% for resilient and ~50% for vulnerable, DFS: ~60% for resilient and ~78% for vulnerable). Control and SI groups had a higher percentage of animals within each group, regardless of resilient and vulnerable phenotype, that could successfully reach criterion (Control: ~80% for both resilient and vulnerable phenotypes; SI: ~90% for both resilient and vulnerable phenotypes) (F_1,22_ = 0.3121; *p* = 0.582) (Figure 4D). Interestingly, all animals within each group that learned the task were able to complete their first successful crossing PLP from criterion (position 4) in 1 trial. When measuring successful crossing rates of animals that learned within each group, there were no differences between the resilient and vulnerable phenotypes, and ANOVA did not reveal significant differences in Group × Treatment interaction (F_3,54_ = 0.8704; *p* = 0.4622), but the Control group had the lowest overall pass rates, and Tukey’s post hoc analysis revealed significant differences in the Control resilient group (*p* < 0.01 for all comparisons) (Figure 4E). When measuring success rates from criterion, there were no differences between the resilient and vulnerable phenotypes, and ANOVA did not reveal significant differences in Group × Treatment interaction (F_3,54_ = 1.127; *p* = 0.3463), but the Control group had the lowest percent success rates from criterion than any other treatment group, and Tukey’s post hoc analysis revealed this was significant in the Control resilient group (*p* < 0.001 for all comparisons) (Figure 4F). This was not due to an observable lack of ability or impairment, but attributed to the exploratory and maintenance behaviors these animals engaged in after learning. Interestingly, Control resilient rats had lower success rates compared to Control vulnerable rats. When comparing the instances of additional behaviors exhibited between resilient and vulnerable phenotypes within each group, it was found that resilient animals on average exhibited a higher instance of the off-task behaviors mentioned above compared to vulnerable animals (Supplementary Table S1).

Additionally, when measuring the average time to complete a trial during LP and PLP, the Control and SI resilient and vulnerable groups had relatively similar completion times for each. The SR and DFS groups had longer completion times than the Control and SI groups during the LP, but the SR reduced their completion time from LP by ~40 s and ~30 s, respectively. During the LP, 100% of animals in each group failed a trial at least once. Of the total number of fails in each group, most were due to the animal falling off the beam rather than the animal timing out during the trial (Table 3). During the PLP, the total number of failures in each group increased. Interestingly, most failures in the SI and DFS groups were due to falling off the beam rather than the animal timing out, indicating they attempted to traverse the beam but were unable due to higher instances of disequilibrium; however, Control and SR group failures were due to the animal timing out during the trial rather than falling off the beam (Table 4). Comparing their behaviors during the PLP, the Control group’s timing out was due to increased exploratory and maintenance behaviors, while the SR group’s timing out appeared to be related to increased fear and disequilibrium, which was associated with them not moving from their start position.

## 4. Discussion

In this study, we assessed the effects of SI and SR on motor learning, sensorimotor function, and behavior during the BB task, and how these effects may vary with stress resilience and vulnerability based on stress-induced changes in sleep. We found both sensorimotor and behavioral deficits associated with SR and unexpected positive effects of SI on performance in the BB task, and these interactions differed depending on sleep-related stress resilience and vulnerability. However, the interactions among stressors and individual differences in stress responsivity are complex and involve more than simple alterations in motor ability that may affect performance. Performance on the BB task requires the use and integration of multiple neural systems, including those subserving sensorimotor function, learning and memory, cognition (including attention, perception, understanding, and decision making), emotion, and likely others. In this study, we also showed that performance in the BB task was heavily influenced by learning ability and behavioral sensitivity and/or flexibility.

The consequences of single or compound inflight stressors on one or more of these systems varied with stress resilience and vulnerability. For example, SI appeared to have a greater effect on learning new gross motor skills, as both SI resilient and vulnerable animals exhibited a temporary blunted ability to learn in the beginning of the BB task, and this effect was exacerbated in SI vulnerable rats. Previous reports in mice [28,29] and fish [30] have shown that SI can impair hippocampal neurogenesis and learning skills, and long-term SI can result in morphological changes in the hippocampus, specifically atrophy of the CA1 area [31]. Social interaction appears to be linked to learning and is evolutionarily conserved across taxonomic classes. The hippocampus is a key region for both memory and sleep [32,33], and stress and/or stress-induced changes in sleep, can both alter the processing of new information [34] and the retrieval of previously acquired memories [35]. This evidence supports our observed learning impairment in the SI animals during the beginning of the BB task, and it is plausible that the increased stress sensitivity of the SI vulnerable animals led to a greater learning impairment, as stress can alter hippocampal morphology and induce synapse loss within the hippocampus [36,37]. These animals did exhibit increased disequilibrium compared to Controls, but this did not hinder SI animals’ successful learning and completion of the BB task from criterion, regardless of resilient or vulnerable classification, as nearly 100% of animals of each phenotype learned the task with almost 100% accuracy. Overstimulation of adrenergic components in brain, specifically those connecting the hippocampus, hypothalamus, and brainstem is implicated in inducing disequilibrium [38]. This neural circuit is also intimately related to sleep regulation [39]. While little is known about the neural consequences of SI, this work suggests that neural regions that regulate sleep can also modulate sensorimotor function, potentially through hippocampal pathways.

Contrastingly, SR impaired gross motor performance needed for the BB task in some individuals that hindered their ability to traverse the beam and required the highest number of trials to reach criterion. These sensorimotor problems are consistent with previous reports [3,4,5,40] and were exacerbated in the vulnerable phenotype. Combined, this resulted in one of the lowest percent of animals that reached criterion of any treatment group. SR animals also displayed a high frequency of fear behavior not seen in Control and SI animals, including fearful or anxious and freezing behaviors. This was greater in vulnerable animals, suggesting SR may increase sensitivity to stress, leading to an exaggeration of fear and related behaviors that may also impair initial learning, as SR vulnerable, but not resilient, animals exhibited learning impairments similar to SI animals during the beginning of the BB task. Motor skills needed for the BB task may also be hindered by the ability to appropriately cope with stress [34], or conversely, impaired motor skills may impact emotion. Several studies have characterized the negative effects of SR on hippocampal functionality [41,42,43], while others have shown that SR can affect the hypothalamus, specifically monoamine metabolism—for example, dopamine. Recent evidence has shown that dopamine is important for motor learning in multiple species, and dopamine-dependent learning mechanisms are only active during sensorimotor adaptation [44]. Therefore, it is possible that sensorimotor deficits induced by SR also act through similar neural systems to those discussed above for SI, possibly through dopaminergic signaling pathways.

Interestingly, when exposed to DFS, the initial learning deficits observed in the groups that received SI or SR alone were ameliorated. This was unexpected, as we hypothesized this combination would exacerbate any deficits or complications observed in SI or SR exposure alone. To our knowledge, these interactions have not been reported before. The effects of SI alone are somewhat understood, but acute and chronic SI appear to have differential outcomes, namely on responses to stimuli and alterations in neural circuits [45,46]. However, a deficit in social interactions, acute or chronic, have been shown to increase seeking/craving of future interactions that may alter other behaviors [45,46], leading some to suggest shorter periods of SI can be beneficial and lead to an increased ability to focus. Our findings suggest that this putative beneficial effect on learning and memory may occur even with chronic SI, and these effects can change when in combination with SR. DFS animals still exhibited high levels of disequilibrium and fear, causing them to fail to complete the task, or not even attempt the task altogether, leading to similar percent success rates as SR animals, suggesting that SR has a greater impact on systems that regulate motor ability and emotional processing compared to SI, and that SI is not able to ameliorate the effects of SR on these systems that were observed for learning and memory. Alterations in freezing associated with irradiation, including increases, have been previously reported [47]. Thus, it is possible that greater freezing in these groups reflects less behavioral flexibility, rather than greater actual fear (or perhaps a combination of reduced behavioral flexibility and increased fear). Additional work will be required to determine whether and how SR differentially impacts neural activity in resilient and vulnerable rats and its relevance.

The Control group’s behaviors and approach to the BB task were categorically different from those of the SI, SR, and DFS groups. Following learning, their engagement in more exploratory and maintenance off-task behaviors later in the BB task was associated with higher rates of timing out and an unwillingness to continue to do the task required of them. Executive functions play an important role in regulating behavior and impulses, and in processing and regulating affect, motivation, and arousal [48]. It is possible these animals may have reduced motivation to continue the repetitive BB task and take advantage of opportunities to engage in other behaviors over time. Exposure to SI or SR may alter executive functions, which might reduce the behavioral flexibility or modify motivation when given a task. This likely is also influenced by changes in emotion and motor ability. Control vulnerable animals exhibited off-task behaviors, though they were less frequent than in their resilient counterparts, leading to a higher percent success rate. This may be attributed to the increased emotional sensitivity in vulnerable animals that made them less behaviorally flexible and more focused on completing the task compared to the resilient Control animals. Thus, differences between resilient and vulnerable rats may reflect complex interactions between motivation and emotion that impact cognitive and behavioral flexibility in ways that would be important for astronaut performance.

Overall, our study demonstrated the single and compound effects of SI and SR on learning and memory and sensorimotor performance, and that SI and SR interactions can be assessed behaviorally. Because we found that standard behavioral measures on the BB test were inadequate to fully assess the effects of SI and SR, we developed additional assessments of the animals’ behavioral repertoires. These assessments demonstrated both motor deficits/changes and interactions between motor performance and emotion and also illustrated that motor responses do not provide a full explanation for altered BB performance effects. Moreover, we found that resilient and vulnerable rats maintain their phenotypes under SI and SR, and that vulnerable rats showed greater deficits on some measures. Unfortunately, the resilient and vulnerable phenotypes can vary across cohorts; thus, it is virtually impossible to ensure equal distributions across groups. This led to low power in some of our statistical analyses; however, differences can still clearly be observed and suggest that this model may be useful for determining the role of individual differences in the effects of SI and SR.

The need to understand individual differences in the ability to cope with stress and also the effects of heterogeneous stressors is being widely recognized [49]. In addition to the present finding on the greater impact of SR on emotion and sensorimotor function, individual differences in the impact of SR on performance have been demonstrated in executive function and attention in rats, with 32–36% (attentional set-shifting test performance) and 40% (rodent psychomotor vigilance test) of rats performing the tasks poorly [40,50]. This suggests multi-system effects of SR and potentially other stressors can vary with individual resilience and vulnerability. The unexpected outcomes of SI and SR interactions also highlight the need for more detailed mechanistic studies that reveal the neural consequences of the single and compound stressor exposure. However, understanding both the effects of stressors and how they differ across individuals will be of particular importance for astronauts who will be subjected to chronic and/or intermittent stressors in a unique environment that can impact their mental and physical health, as well as their ability to perform mission related tasks.

## Figures and Tables

**Table 1:** **Observable Behaviors During the Learning Period (LP) of the Balance Beam Task Differed Between Treatment Groups.** Table showing the percent occurrence of the total behavioral categories exhibited during the LP of the balance beam task. Behaviors within each category include the following: Exploratory—vacillation, spinning, and rearing; Disequilibrium—compromised ambulation, impaired gait, balance issues, and disorientation; Fear—freezing; and Maintenance—grooming. Compared to Control: ++ *p* < 0.01, +++ *p* < 0.001.

LP	Exploratory	Disequilibrium	Fear	Maintenance	Total Instances
Control	97.48%	0.00%	0.00%	2.52%	119
SI	57.14%, +++	42.86%	0.00%	0.00%	14
SR	10.64%, +++	79.79%, +++	6.38%	3.19%	94
DFS	18.87%, +++	73.58%, ++	5.66%	1.89%	53

**Table 2:** **Observable Behaviors During the Post-Learning Period (PLP) of Balance Beam Task Differed Between Treatment Groups.** Table showing the percent occurrence of the total behavioral categories exhibited during the PLP of the balance beam task. Behaviors within each category include the following: Exploratory—vacillation, spinning, and rearing; Disequilibrium—compromised ambulation, impaired gait, balance issues, and disorientation; Fear—freezing; and Maintenance—grooming. Compared to Control: + *p* < 0.05, ++ *p* < 0.01, +++ *p* < 0.001.

PLP	Exploratory	Disequilibrium	Fear	Maintenance	Total Instances
Control	75.62%	0.00%	0.00%	24.38%	201
SI	56.45%	40.32%, +++	0.00%	3.23%, +	124
SR	9.03%, ++	71.61%, +++	12.90%	6.45%, +	155
DFS	13.37%, ++	81.28%, +++	4.81%	0.53%, +	187

**Table 3:** **Performance in the Balance Beam during the Learning Period (LP).** Table showing the average time to complete a trial, the percentage of animals that failed a trial, the percentage of total failures that occurred due to falling off the beam, the percentage of total failures that occurred due to timing out, and the total number of fails that occurred during the learning period LPof resilient (R) and vulnerable (V) animals within each treatment group during the balance beam task.

LP	Time to Complete a Trial	Animals that Failed	Failed Due to Falling	Failed Due to Timing Out	Total Number of Fails
**Control-R**	46.79 s	100%	92.31%	7.69%	78
**Control-V**	44.72 s	100%	79.66%	20.34%	59
**SI-R**	30.83 s	100%	98.87%	1.13%	89
**SI-V**	30.25 s	100%	100%	0%	71
**SR-R**	71.81 s	100%	96.88%	3.12%	64
**SR-V**	73.40 s	100%	100%	0%	11
**DFS-R**	59.94 s	100%	68.42%	31.58%	19
**DFS-V**	51.96 s	100%	100%	0%	3

**Table 4:** **Performance in the Balance Beam is altered from the LP to Post-Learning Period (PLP).** Table showing the average time to complete a trial, the percentage of animals that failed a trial, the percentage of total failures that occurred due to falling off the beam, the percentage of total failures that occurred due to timing out, and the total number of fails that occurred during the PLP of resilient (R) and vulnerable (V) animals within each treatment group during the balance beam task.

PLP	Time to Complete a Trial	Animals that Failed	Failed Due to Falling	Failed Due to Timing Out	Total Number of Fails
**Control-R**	54.97 s	78.57%	36.67%	63.33%	150
**Control-V**	42.63 s	100%	29.62%	70.37%	81
**SI-R**	37.11 s	82.35%	92.30%	7.70%	91
**SI-V**	27.56 s	16.67%	100%	0%	41
**SR-R**	33.01 s	45.45%	21.50%	78.50%	107
**SR-V**	28.11 s	100%	29.73%	70.27%	37
**DFS-R**	27.01 s	66.67%	52.94%	47.06%	119
**DFS-V**	21.60 s	66.67%	77.78%	22.22%	9

## Data Availability

Experimental data are available upon request.

## Supplementary Materials

The following are available online at https://www.mdpi.com/article/10.3390/life13030826/s1, Figure S1: Sleep Does Not Differ between Control and Sham Groups. Figure S2: Balance Beam Performance Does Not Differ between Control and Sham Groups. Table S1: Resilient Animals Exhibit More Off-Task Behaviors Compared to Vulnerable Animals. Table S2: Summary of Results.
