# Supplementary material for: Differential Impact of Social Isolation and Space Radiation on Behavior and Motor Learning in Rats
Source: Life (Basel). 2023 Mar 18;13(3):826. doi: 10.3390/life13030826 (PMC10057568; doi:10.3390/life13030826)
Supplement: Supplementary file 1 [file life-13-00826-s001.zip › life-2219549-supplementary.pdf]

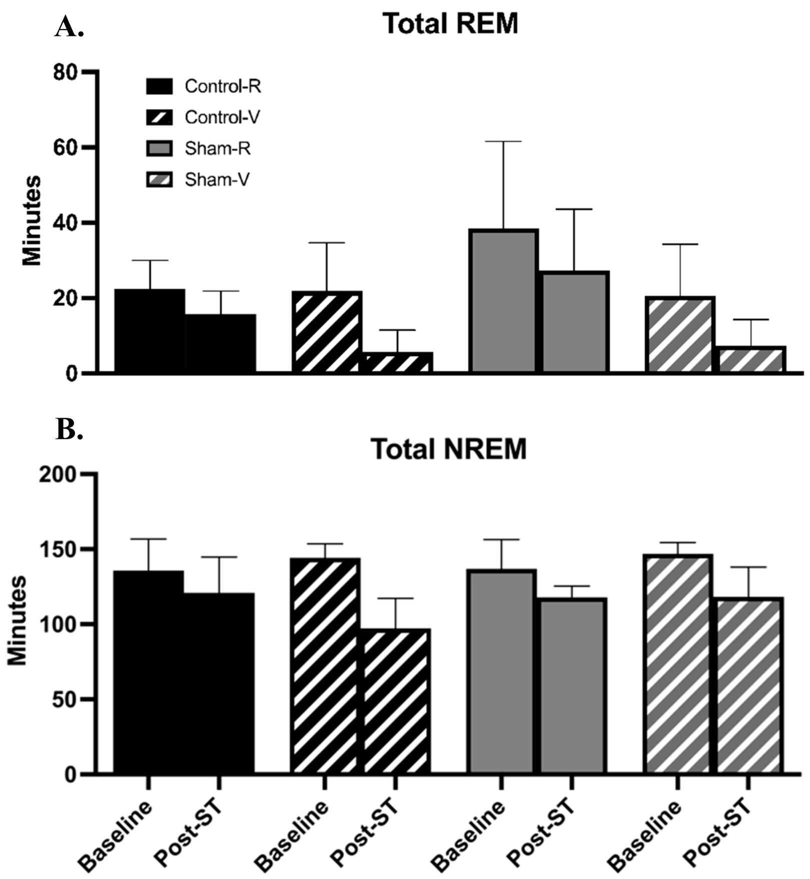

Supplementary Figure S1: Sleep Does Not Differ between Control and Sham Groups. Graphs plotting the first 4 hrs of sleep recording during baseline and after shock training (Post-ST) between Control and Sham resilient (R) and vulnerable (V) animals for **A)** REM ± SEM and **B)** NREM ± SEM. Sham animals traveled with SR treated groups as a control to account for any potential negative effects caused by the transit required for the experiment. Control animals never traveled and remained in their housing rooms on campus.

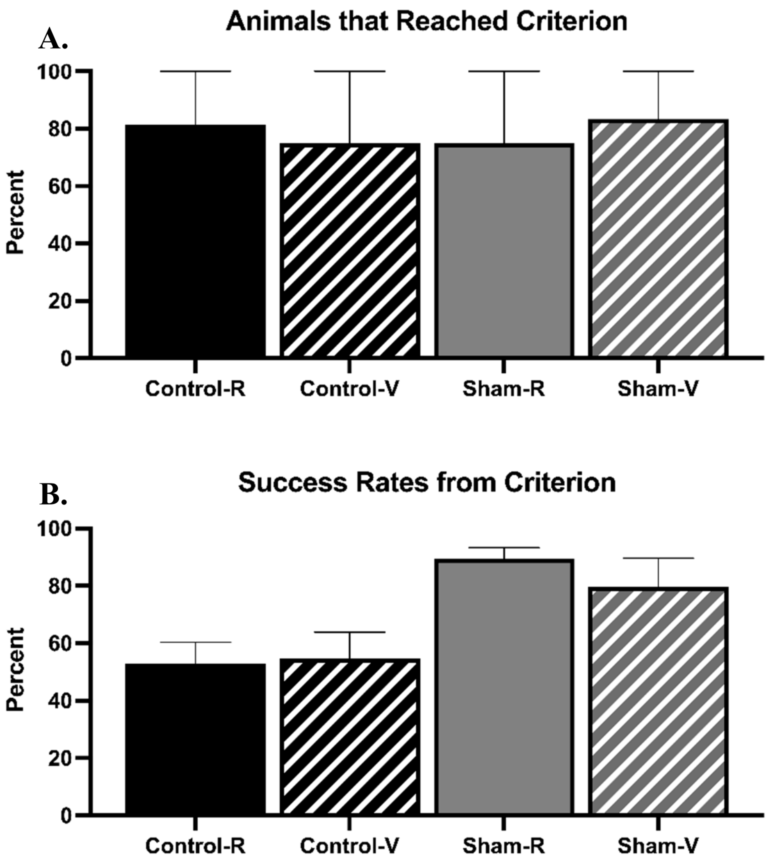

Supplementary Figure S2: Balance Beam Performance Does Not Differ between Control and Sham Groups. Graphs plotting differences between resilient (R) and vulnerable (V) phenotypes within each treatment group for **A)** the percent of animals ± SEM that reached criterion (position 4) and **B)** the percent success rates of animals ± SEM from criterion (position 4).

Supplementary Table S1: Resilient Animals Exhibit More Off-Task Behaviors Compared to Vulnerable Animals. Table showing the percentage between resilient and vulnerable phenotypes of the total behaviors exhibited during the learning period (LP) and post-learning period (PLP) within each treatment group during the balance beam task.

	**LP**		**PLP**	
	**Resilient**	**Vulnerable**	**Resilient**	**Vulnerable**
**Control**	82.99%	17.01%	64.53%	35.47%
**SI**	57.14%	42.86%	71.40%	28.60%
**SR**	93.08%	6.92%	85.59%	14.41%
**DFS**	75.68%	24.32%	84.83%	15.17%

Supplementary Table S2: Summary of Results. Table showing a summary of the overall results between resilient and vulnerable phenotypes within each treatment group during the balance beam task.

	**Total Instances of Disequilibrium During BB Task**		**Total Instances of Fear Behavior During BB Task**		**Number of Times an Animal Neglected to Attempt a Trial During BB Task**		**Percent of Animals that Reached Criterion**	
	**Resilient**	**Vulnerable**	**Resilient**	**Vulnerable**	**Resilient**	**Vulnerable**	**Resilient**	**Vulnerable**
**Control**	0	0	0	0	0	0	80%	80%
**SI**	75	13	0	0	0	0	90%	90%
**SR**	277	20	7	18	114	26	75%	50%
**DFS**	230	53	13	2	70	2	60%	78%
